# Comparison of Flow Injection-MS/MS and LC-MS/MS for the Determination of Ochratoxin A

**DOI:** 10.3390/toxins13080547

**Published:** 2021-08-06

**Authors:** Kai Zhang

**Affiliations:** Office of Regulatory Science, Center for Food Safety and Applied Nutrition, U.S. Food and Drug Administration, 5001 Campus Drive, HFS-717, College Park, MD 20740, USA; kai.zhang@fda.hhs.gov; Tel.: +1-240-402-2318

**Keywords:** flow injection, LC-MS-MS, ochratoxin A

## Abstract

Two methods for measuring ochratoxin A in corn, oat, and grape juice were developed and compared. Flow injection (FI) and on-line liquid chromatography (LC) performances were evaluated separately, with both methods using a triple quadrupole tandem mass spectrometer (MS/MS) for quantitation. Samples were fortified with ^13^C uniformly labeled ochratoxin A as the internal standard (^13^C-IS) and prepared by dilution and filtration, followed by FI- and LC-MS/MS analysis. For the LC-MS/MS method, which had a 10 min run time/sample, recoveries of ochratoxin A fortified at 1, 5, 20, and 100 ppb in corn, oat, red grape juice, and white grape juice ranged from 100% to 117% with RSDs < 9%. The analysis time of the FI-MS/MS method was <60 s/sample, however, the method could not detect ochratoxin A at the lowest fortification concentration, 1 ppb, in all tested matrix sources. At 5, 20, and 100 ppb, recoveries by FI-MS/MS ranged from 79 to 117% with RSDs < 15%. The FI-MS/MS method also had ~5× higher solvent and matrix-dependent instrument detection limits (0.12–0.35 ppb) compared to the LC-MS/MS method (0.02–0.06 ppb). In the analysis of incurred corn and oat samples, both methods generated comparable results within ±20% of reference values, however, the FI-MS/MS method failed to determine ochratoxin A in two incurred wheat flour samples due to co-eluted interferences due to the lack of chromatographic separation.

## 1. Introduction

Ochratoxin A is a toxic metabolite produced by fungi of the genera *Aspergillus* and *Penicillium* and is often detected in various food commodities, including tree nuts, cereal grains, grape juice, coffee beans, and dry fruits [1,2,3,4,5,6]. Ochratoxin A is of significance from a public health point of view [7,8,9,10] because long-term dietary intake of foods contaminated by ochratoxin A has been linked to adverse health effects in different populations, including nephrotoxic, hepatotoxic, embryotoxic, teratogenic, neurotoxic, immunotoxic, genotoxic, and potentially carcinogenic effects [11,12,13]. Furthermore, ochratoxin A could also act synergically with other co-occurring mycotoxins in foods and feeds, posing a potential threat to humans and animals [14,15,16].

As complete removal of ochratoxin A through current production and processing methods of agricultural products is not feasible [2,17], to minimize dietary exposure, regulatory bodies worldwide have established maximum levels of ochratoxin A [18,19,20,21]. In the U.S., the Food and Drug Administration (FDA) has been monitoring the occurrence of ochratoxin A in imported and domestic foods and requires the reporting of regulatory samples above 20 ppb ochratoxin A for risk assessment [22].

To comply with established regulatory limits, the analysis of ochratoxin A has become an essential part of international trade, routine mycotoxin monitoring, and regulatory surveillance. A variety of methods have been developed for the determination of ochratoxin A in foods and feeds using enzyme-linked immunosorbent assays (ELISAs) [23], lateral flow [24], aptamer-based biosensors [25], thin layer chromatography (TLC) [26], liquid chromatography (LC) with fluorescence detection (FLD) or ultraviolet/diode array detection (UV/DAD) [27,28], liquid chromatography with tandem mass spectrometry (LC-MS/MS), and high-resolution spectrometry (LC-HRMS) [29,30]. Modern LC-MS/MS offers superior specificity and sensitivity compared to non-mass selective detectors. In addition to chromatographic separation, target analytes are further separated from matrix components using pre-determined precursor/product ion pairs with unique structural characteristics. Furthermore, in LC-MS-based methods, conventional concentration, solid phase extraction (SPE), and/or immunoaffinity cleanup procedures have been gradually replaced by sample dilution for select matrices, simplifying sample preparation and making sample analysis more time efficient and less laborious.

To further improve throughput, an alternative approach to LC-MS-based methods is flow injection tandem mass spectrometry (FI-MS/MS) in which samples are directly introduced into the ionization source without chromatographic separation of target analytes, significantly increasing the speed of sample analysis. In the past, such an approach suffered from poor sensitivity, ion suppression, and interferences of isobaric compounds in the matrix [31]. With the continuous improvements in ionization and detection technologies, MS can identify target analytes in a variety of biological, environmental, and food matrices without LC, where matrix effects can be mitigated using large dilution factors. In recent years, FI-MS/MS has been increasingly used in the areas of metabolomics, lipidomics, pharmaceuticals, and clinical and environmental analysis [32,33,34,35], but its application for food analysis, especially mycotoxin analysis, has been only marginally explored [31,36]. If FI-MS/MS could provide comparable results to LC-MS/MS, it would become another valuable tool for mycotoxin analysis. The primary goal of this study was to evaluate and compare FI-MS/MS and LC-MS/MS for the determination of ochratoxin A in corn, oat, and juice samples prepared by solvent extraction and dilution. The performance (quantitation, identification, limit of quantification, and matrix effects) of the two methods was evaluated in representative matrices such as cereal grains and grape juices.

## 2. Results and Discussion

The main incentive to explore FI-MS/MS is the speed of analysis. In this study, the analysis time for every sample was less than 60 s. Compared to LC-MS/MS (10 min/sample), this would be a much more time-efficient approach for routine sample analysis, especially for laboratories that need to analyze a large number of samples on a regular basis, although the lack of chromatographic separation leads to more matrix entering the mass spectrometer and potentially more instrument maintenance. However, throughput is just one of the key factors that determine the suitability of a method for mycotoxin analysis. Compared to LC-MS/MS, FI-MS/MS bypasses an LC separation and directly infuses samples into the ion source, a practice which could affect chromatographic characteristics (peak shape, height), enhance matrix effects, and cause isobaric interferences that would be otherwise separated by LC. Therefore, in the following discussion, we aim to assess the impact of FI-MS/MS on recovery, sensitivity, and matrix effects using LC-MS/MS as the benchmark.

### 2.1. Recovery Study

Of particular importance is the comparison of recoveries of both methods at different concentrations in selected matrices: grape juice, oat, and corn. Independent preparations of spiked samples containing four replicates of each target concentration (1, 5, 20, and 100 ppb) were analyzed by FI-MS/MS and LC–MS/MS. The measured recoveries and relative standard deviations (RSDs) are presented in Table 1. At concentrations of 5, 20, and 100 ppb, the FI-MS/MS method had average recoveries ranging from 79 to 117% with RSD 2–15%. However, ochratoxin A was not detected at 1 ppb across the four matrices by FI-MS/MS. The LC-MS/MS method not only achieved the detection of ochratoxin A at 1, 5, 20, and 100 ppb, but also demonstrated a smaller variability among recoveries (100–117%) with RSD 2–8%. As the FI-MS/MS method failed to detect ochratoxin A at 1 ppb, we speculated that the root cause was insufficient sensitivity caused by ion suppression. If it were due to sample preparation (e.g., poor extraction efficiency) then the LC-MS/MS method would similarly not detect ochratoxin A at 1 ppb.

### 2.2. Instrument Limit of Quantitation

To further understand why FI-MS/MS failed to detect ochratoxin A in the spike samples at 1 ppb, we determined the solvent- and matrix-dependent instrument limit of quantitation. Table 2 summarizes the instrument LOQ results of ochratoxin A for FI-MS/MS and LC-MS/MS in oat, corn, grape juice, and solvent. As expected, the instrument LOQ for FI-MS/MS, ranging from 0.12 to 0.35 ppb, was much higher than LC-MS/MS, ranging from 0.02 to 0.06 ppb.

### 2.3. Evaluation of Matrix Effects

For MS-based analysis, ionization of target analytes is prone to matrix effects, signal enhancement, or suppression. In ESI, ion suppression is often observed due to insufficient ionization of target analytes, leading to the loss of sensitivity. As expected, FI-MS/MS would suffer more signal suppression than LC-MS/MS because there would be more co-eluted matrix components competing with ochratoxin A for charge in the ion source than LC-MS/MS, in which the LC could chromatographically separate ochratoxin A and matrix components by the difference in their interactions with the mobile and stationary phases on the column.

**Figure 1:**
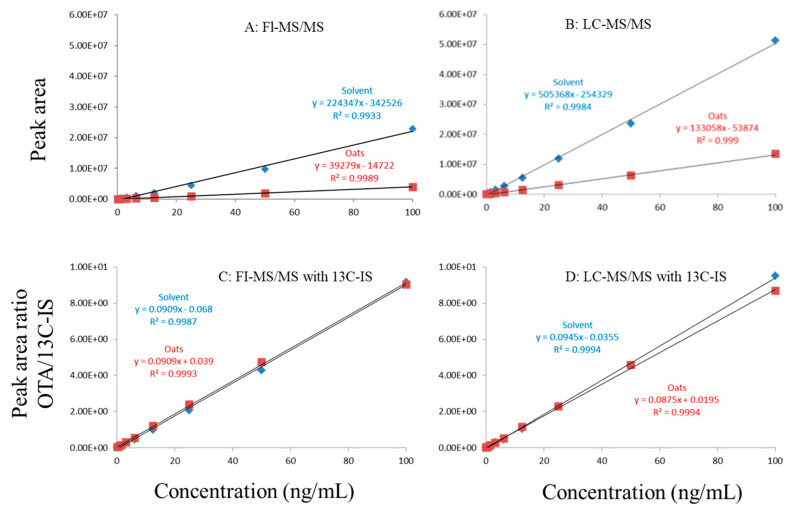
Matrix effects of in oat. (**A**) FI-MS/MS; (**B**) LC-MS/MS; (**C**) FI-MS/MS with 13C-IS; (**D**) LC-MS/MS with 13C-IS.

In this study, matrix effects of the FI-MS/MS and LC-MS/MS methods were evaluated using responses of ochratoxin A at different concentrations in solvent and matrix extracts as well as solvent-only and matrix-matched calibration curves. The corresponding slope coefficients and peak areas/heights of ochratoxin A at concentrations 0.1–100 ppb were monitored and compared. As shown in Figure 1A,B, visually, there were obvious difference between the oat matrix-matched calibration curves and solvent-only calibration curves because, within the concentration range, the responses of ochratoxin A at the same concentrations in the solvent are much higher than that in the matrix. Additionally, the incorporation of LC improved the peak shape of ochratoxin A, further improving quantitation. For example, at 0.5 ppb, the peak height of ochratoxin A was above 80,000 (absolute unit) on the LC column while, without the column, the peak height barely reached 30,000. 

**Figure 2:**
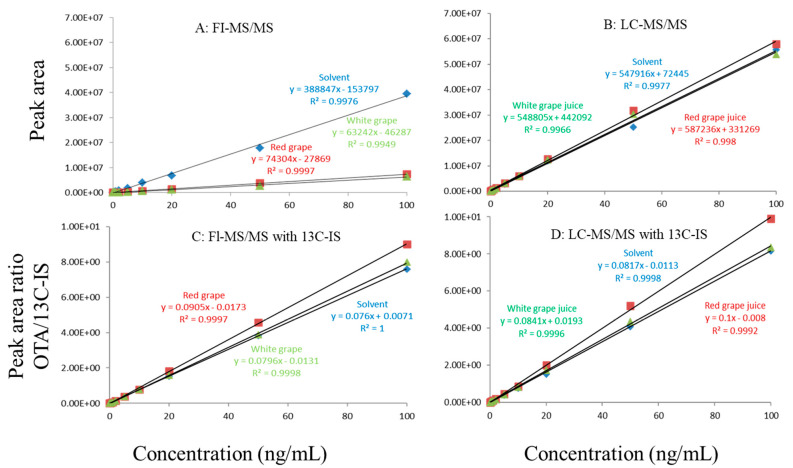
Matrix effects in red and white grape juice. (**A**) FI-MS/MS; (**B**) LC-MS/MS; (**C**) FI-MS/MS with 13C-IS; (**D**) LC-MS/MS with 13C-IS.

**Figure 3:**
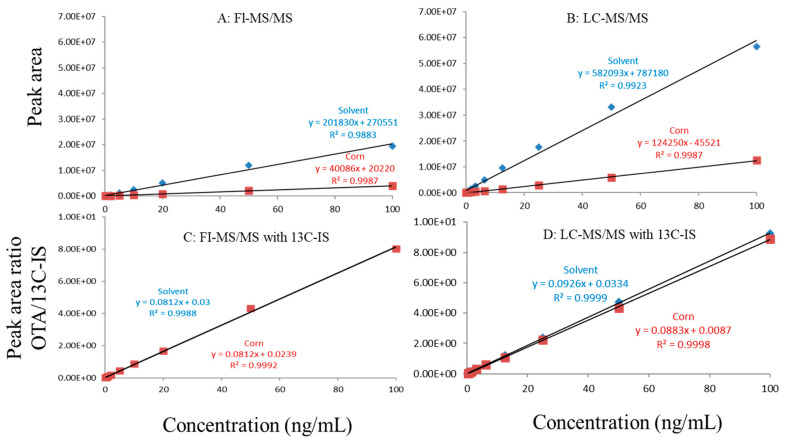
Matrix effects in corn. (**A**) FI-MS/MS; (**B**) LC-MS/MS; (**C**) FI-MS/MS with 13C-IS; (**D**) LC-MS/MS with 13C-IS.

Regardless of whether FI-MS/MS or LC-MS/MS was used, in all tested matrices, the slopes of matrix-matched calibration curves were much smaller than the corresponding solvent-only calibration curves (Figure 1A,B, Figure 2A,B and Figure 3A,B), demonstrating the negative impact of matrix components on the ionization of ochratoxin A. For example, Figure 1A shows that the response of ochratoxin A at 1, 10, and 100 pbb in the solvent using FI-MS/MS was almost 9× higher than that in the oat matrix, suggesting if one ignored matrix effects and used solvent-only calibration curves for quantitation without internal standard correction, concentrations of ochratoxin A in oat samples could be significantly underestimated. There were similar observations associated with the LC-MS/MS method (Figure 1B), though the matrix effects were less pronounced, as chromatographic separation leads to less co-eluted matrix components competing for charges in the ionization source. Similar observations are noted in corn (Figure 2A,B) and grape juice matrices (Figure 3A,B).

To offset matrix effects, it is a common practice to use matrix-matched calibration curves for quantitation. Alternatively, one could take advantage of stable isotope dilution for quantitation, avoiding the preparation of matrix-matched calibration standards. In this study, we used ^13^C-ochratoxin A as the internal standard and spiked it into samples prior to extraction. This enabled the compensation of the loss of ochratoxin A during sample preparation and/or ion suppression caused by co-eluted matrix components. Signal suppression was shown to vary between individual matrices, as shown in Figure 1A,B, Figure 2A,B and Figure 3A,B. Using ^13^C-ochratoxin A as the internal standard, matrix-matched and solvent-only calibration curves would generate comparable quantitative results (Figure 1C,D, Figure 2C,D and Figure 3C,D). For example, in oat, grape juice, and corn matrices, the comparison of both methods showed that the difference in the signal ratio between ochratoxin A and ^13^C-ochratoxin A between the two calibration curves was less than 10%, at 1, 10, and 100 ppb. Without chromatographic separation, ion suppression of ochratoxin A by FI-MS/MS was more pronounced than LC-MS/MS (Figure 1, Figure 2 and Figure 3), leading to a higher limit of quantification and more narrow linear dynamic range (Table 3). At high concentrations, such signal suppression could be offset by using ^13^C-ochratoxin A as the internal standard but, at low concentrations, such suppression would impact the detectability of ochratoxin A. In other words, ^13^C-IS cannot physically eliminate matrix-induced ion suppressions, though it offers quantitative correction when ochratoxin A meets the criteria for identification. 

### 2.4. Analysis of Incurred Samples

**Figure 4:**
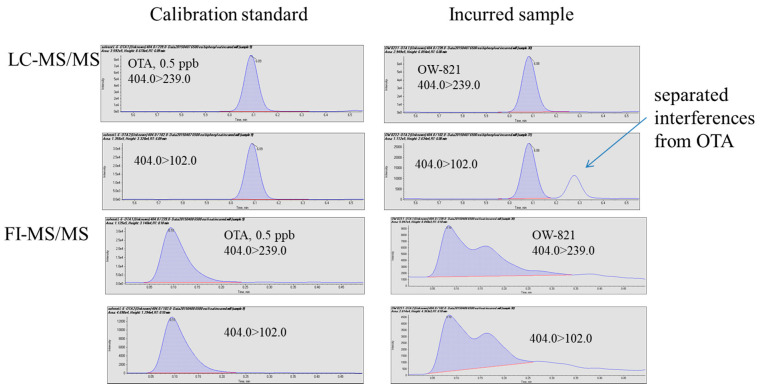
Analysis of an incurred sample using FI-MS/MS and LC-MS/MS.

Several incurred samples and one reference material were analyzed using the two methods. For NIST SRM 1565 (corn), C-3023 (oat), and C-9999 (corn), both methods detected and quantitated ochratoxin A with comparable results (Table 3), which are in good agreement with the reference values with differences less than 20%. In the two wheat flour samples, the LC-MS/MS method quantitated ochratoxin A at 7.6 ± 1.0 and 90.8 ± 3.7 ppb (*n* = 3), respectively. Compared to the corresponding reference values, 7.0 ± 1.8 and 93.7 ± 9.6 ppb, the LC-MS-MS results demonstrated adequate accuracy (difference between measured values and reference values < ±10% of reference values) and precision (RSDs < 15%). The FI-MS/MS method could not quantitate ochratoxin A in the two wheat flour samples. Figure 4 clearly illustrates that co-eluted isobaric interferences made quantitation impossible because of the lack of chromatographic separation, while such interference appeared to be separated from ochratoxin A by the LC. Without interferences, FI-MS/MS could likely achieve acceptable quantitation within a much shorter time, but in the presence of interferences, FI-MS/MS could generate false negatives.

## 3. Conclusions

To eliminate the interferences, in-depth cleanup could be used; however, such an approach could contradict the purpose of FI-MS/MS. One would prefer not to use a time-consuming sample preparation as part of analysis aimed for high throughput, particularly a cleanup that would likely not be automated. Compared to LC-MS/MS methods, FI-MS/MS methods do not involve the selection and evaluation of phase chemistry, pore size, particle size, column length, and internal diameter of LC columns, simplifying the method development and validation. However, these parameters closely interact with MS to determine sensitivity and selectivity of the target analyte, making simple sample preparation such as dilute-and-shoot more compatible for MS analysis. With LC columns, these key parameters could be used to purify analytes, remove matrix effects, and increase selectivity, as shown in the above discussion. 

Seemingly, the benefits of LC are less appreciated as MS becomes more sensitive and selective. In this study, even when sensitivity was not an issue, co-eluted interferences prevented the identification and quantitation of ochratoxin A, a fact which suggests that one should not underestimate the limitation associated with FI-MS/MS while trying to simplify LC-MS/MS analysis. Additionally, FI-MS/MS has competing high-throughput technologies. RapidFire mass spectrometry can introduce samples at a rate of 5–10 s/sample [37] and acoustic mist ionization mass spectrometry shortens the time to 0.3 s/sample [38,39]. The future outlook of FI-MS/MS relies on more sensitive and selective MS, faster sample introduction systems, and, particularly, sample cleanup procedures that could minimize interference from the matrices, likely one of the most challenging issues that will continue to hinder the application of FI-MS/MS for complex food matrices.

## 4. Materials and Methods

Ochratoxin A stock solution (10 ppm) and ^13^C_20_-ochratoxin A (10 ppm) were purchased from Romer Labs, Inc. (Newark, DE, USA). LC grade acetonitrile, water, and MS grade formic acid and ammonium formate were purchased from Thermo Fisher Scientific, (Waltham, MA, USA). A working solution of ^13^C_20_-ochratoxin A (^13^C-IS) was prepared at 500 ppb in acetonitrile. Calibration solutions of ochratoxin A ranging from 0.01 to 1000 ppb were prepared by a series of dilutions of the stock solution using acetonitrile. An incurred corn reference material (SRM 1565) was purchased from NIST (Gaithersburg, MD, USA). A second incurred corn sample (MT-C-9999G) was purchased from Trilogy Analytical Laboratory (Washington, MO, USA). Two incurred wheat flour samples (OW-821 and OW-825) and one incurred oat sample (C-3023) were purchased from Romer Labs (Newark, DE, USA).

### 4.1. Sample Preparation

Samples were prepared via water slurry, extraction, centrifugation, and filtration [40]. Samples (25.0 ± 0.5 g each) were weighed out in a 100 mL disposable grinding chamber and blended with 25.0 ± 0.5 g of water (HPLC grade) for 1.5 min at 25,000 rpm using an IKA Tube Mill. A test portion (2.00 ± 0.05 g, equivalent to 1.00 g of sample on a dry basis) from the blended sample was transferred into a 15 mL disposable screw-capped polypropylene centrifuge tube and fortified with 40 μL of the ^13^C-IS working solution. The tube was re-capped and vortexed for 30 s. Extraction solvent (4.0 mL; acetonitrile/water, 50/50, *v*/*v*) was added into the tube and tubes were placed on a shaker with pulsation (Glas-Col, Terre Haute, IN, USA) for 30 min at a speed set to 80 and pulser frequency set at the middle mark of the dial (~30–35 pulsations/min). Samples were centrifuged for 15 min at 4500 rpm (*rcf 4200*) ThermoElectro Corp., Milford, MA, USA) and the extraction supernatant (~1.0 mL) was pushed through a 0.2 µm PTFE filter. The resulting filtrates were transferred into autosampler vials for FI- and LC-MS/-MS analysis. Red and white grape juice samples were prepared without the water slurry step. Juice samples (1.00 ± 0.05 g each) were weighed into 15 mL disposable screw-capped polypropylene centrifuge tubes and fortified with 40 mL of the ^13^C_20_-ochratoxin A working solution, followed by the addition of 10 mL extraction solvent. Then, samples were shaken, centrifuged, and filtered as described above. 

### 4.2. Recovery Studies

Recovery studies were carried out using spiked oat, corn, and red and white grape juice at 1, 5, 20, and 100 ppb. At each concentration, samples were prepared in quadruplicate. The concentrations of ochratoxin A in spike samples was determined using solvent-only calibration curves and the peak area response ratio of ochratoxin A to that of 13C-IS. Each calibration curve consisted of multiple calibration standards and was constructed using least squares regression. 

### 4.3. LC-MS/MS Analysis

Samples were analyzed using a Shimadzu Prominence/20 series (Columbia, MD, USA) liquid chromatograph coupled with an SCIEX (Forest City, CA, USA) 6500 quadruple linear ion trap (QTrap) mass spectrometer equipped with an electrospray ionization (ESI) interface source. LC separation was achieved on a Phenomenex Kinetex biphenyl 100A column (100 mm × 2.1 mm i.d., 2.6 μm) with a 2 mm × 2.1 mm i.d. guard cartridge (Torrance, CA. USA). The stationary phase is biphenyl with TMS endcapping. The LC mobile phases consisted of 10 mM ammonium formate/0.1% formic acid/water (A) and 10 mM ammonium formate/0.1% formic acid/methanol (B). Gradient elution at 0.5 mL/min flow rate was initiated at 5% B, ramped to 60% B in 2 min via linear gradient mode and then to 100% B in 5 min via exponential gradient mode (pump B curve 3 to 6), held for 2 min, and changed to 5% B at 7.5 min. Total run time was 10 min including 2.5 min of column conditioning time. The injection volume was 5 μL, and the column temperature was set at 40 °C. Ionization source-dependent parameters in positive ionization mode were set as follows: curtain gas (CUR), 30 psi; ion spray voltage, 4500 V; nitrogen collision gas (CAD), medium; source temperature (TEM), 450 °C; ion source gases 1 and 2 (GS1 and GS2), at 50 and 60 psi. Resolution at Q1 and Q3 were set to unt. Retention time, DP, EP, CE, and CXP, and the two specific, selected MRM transitions of ochratoxin A and ^13^C-IS are listed in Table 4 and were used for data acquisition. Analyst 1.6 and MQ 2.0 (SCIEX) were used for data processing. Microsoft Excel 2010 (Microsoft, Redmond, WA, USA) was used to calculate recoveries and relative standard deviation (RSD). Identification was achieved following FDA Guidance for Industry 118: Mass Spectrometry for Confirmation of the Identity of Animal Drug Residues [41].

### 4.4. Flow Injection MS-MS Analysis

Samples were directly injected using the Shimazu LC autoinjector into the 6500 without any LC column. Flow rates (0.3, 0.4, 0.5, and 1.0 mL/min) and mobile phase (5%, 50%, and 100% methanol with 10 mM ammonium formate and 0.1% formic acid) were compared. The flow rate was set at 0.5 mL/min. Methanol with 10 mM ammonium formate and 0.1% formic acid was used as the mobile phase. Injection volume was 5 µL. MS parameters were the same as those used for LC-MS/MS analysis described above. The total run time for each sample was 0.5 min. 

### 4.5. Matrix Effects

Matrix-matched calibration standards were prepared by spiking ochratoxin A into blank matrix extracts of grape juice, oat, and corn. Blank grape juice, oat, and corn extracts were prepared following sample preparation procedures. ^13^C-IS (10 μL) was fortified into each calibration standard (0.5 mL) prior to FI- and LC-MS/MS analysis. Matrix-matched and solvent calibration standards and curves for ochratoxin A were established. A comparison of signal responses at the same concentrations in solvent and matrix extracts, as well as matrix-matched and solvent calibration curves, was used to demonstrate ionization suppression [42].

### 4.6. Matrix-Dependent Instrument Limit of Detection

The matrix-dependent instrument limits of detection and quantitation (LOD and LOQ, respectively) for ochratoxin A were obtained by following procedures from the U.S. Environmental Protection Agency’s (EPA) protocol [43]. The matrix-dependent LOD was determined by analyzing eight replicates of ochratoxin A solvent and matrix-matched calibration standards, respectively. The mean and standard deviation (SD) of ochratoxin A were obtained from the eight replicates. The matrix-dependent instrument LOD was calculated using the formula LOD = 2.998 × SD (critical t0.01 = 2.998 for degree of freedom (df) of 7). The corresponding matrix-dependent instrument LOQ was calculated as 3 × LOD.

## Figures and Tables

**Table 1:** Average recoveries (RSD, *n* = 4) of FI-MS/MS and LC-MS/MS.

Conc. (ppb)	White Grape Juice		Red Grape Juice		Corn		Oat	
	FI-MS/MS	LC-MS/MS	FI-MS/MS	LC-MS/MS	FI-MS/MS	LC-MS/MS	FI-MS/MS	LC-MS/MS
1	ND	108 (4)	ND	109 (2)	ND	115 (8)	ND	106 (7)
5	84 (6)	106 (2)	80 (4)	105 (3)	89 (7)	109 (5)	117 (15)	103 (3)
20	90 (4)	103 (5)	79 (3)	100 (6)	94 (3)	109 (4)	97 (5)	111 (4)
100	96 (2)	105 (3)	85 (5)	106 (2)	97 (5)	110 (3)	94 (4)	117 (4)

ND: not detected.

**Table 2:** Matrix- and solvent-dependent instrument LOQ, linear range, and linearity of FI-MS/MS and LC-MS/MS for test matrix sources.

Instrument Performance	White Grape Juice		Red Grape Juice		Corn		Oat		Solvent	
	FI-MS/MS	LC-MS/MS	FI-MS/MS	LC-MS/MS	FI-MS/MS	LC-MS/MS	FI-MS/MS	LC-MS/MS	FI-MS/MS	LC-MS-MS
LOQ (ppb)	0.24	0.06	0.28	0.05	0.29	0.06	0.35	0.02	0.12	0.02
Linear range (ppb)	0.25–100	0.05–100	0.25–100	0.05–100	0.25–100	0.05–100	0.5–100	0.02–100	0.1–100	0.02–100
Linearity (*r^2^*)	0.9998	0.9996	0.9997	0.9992	0.9992	0.9998	0.9993	0.9991	0.9992	0.9989

**Table 3:** Analysis of incurred samples by FI-MS/MS and LC-MS/MS (average ± SD ng/g, *n* = 3).

Samples	FI-MS/MS	LC-MS/MS	Ref. Value
C-9999G (corn)	63.0 ± 3.5	62.3 ± 3.2	62.1 ± 17.9
OW-821 (wheat flour)	ND	90.8 ± 3.7	93.7 ± 9.6
OW-825 (wheat flour)	ND	7.6 ± 1.1	7.0 ± 1.8
NIST 1565 blank (corn)	ND	ND	ND
NIST 1565 incurred (corn)	10.8 ± 0.4	8.3 ± 0.3	9.4 ± 1.2
C-3023 (oat)	6.8 ± 1.2	7.6 ± 1.1	8.2 ± 3.6

ND: not detected.

**Table 4:** Molecular weight, formula, and LC-MS/MS parameters of ochratoxin A and ^13^C-ochratoxin A.

Mycotoxin	CAS#	Formula	Molecular Weight (Da)	LC-MS/MS Parameters						
				Rt (min)	Q1 (*m/z*)	Q3 (*m/z*)	DP (V)	EP (V)	CE (V)	CXP (V)
Ochratoxin A	303-47-9	C_20_H_18_ClNO_6_	403.1	6.0	404	239	66	10	41	16
						102	66	10	101	16
^13^C_20_-ochratoxin A	NA	^13^C_20_H_18_ClNO_6_	423.1	6.0	424	250	66	10	41	14
						110	66	10	109	18

## Data Availability

Data available on request following Public Access to Results of FDA-Funded Scientific Research (https://www.fda.gov/science-research/about-science-research-fda/public-access-results-fda-funded-scientific-research, accessed on 3 August 2021).
